# Fibrates for the treatment of cholestatic itch (FITCH): study protocol for a randomized controlled trial

**DOI:** 10.1186/s13063-017-1966-8

**Published:** 2017-05-23

**Authors:** Ruth Bolier, Elsemieke S. de Vries, Albert Parés, Jeltje Helder, E. Marleen Kemper, Koos Zwinderman, Ronald P. Oude Elferink, Ulrich Beuers, the Netherlands Association for the Study of the Liver (NASL) Cholestatic Liver Diseases Study Group, Henk R. van Buuren, Joost P. Drenth, Karel J. van Erpecum, Bart van Hoek, Peter L. M. Jansen, Karin M. van Nieuwkerk, J. Marleen de Vree

**Affiliations:** 1 Tytgat Institute for Liver and Intestinal Research, Department of Gastroenterology and Hepatology, Academic Medical Center, University of Amsterdam, Meibergdreef 9, 1105 AZ Amsterdam, The Netherlands; 2 Liver Unit, Hospital Clínic, IDIBAPS, CIBERehd, Department of Medicine, University of Barcelona, Barcelona, Spain; 3 Department of pharmacy, Academic Medical Center, Amsterdam, The Netherlands; 4 Department of Clinical Epidemiology, Biostatistics and Bioinformatics, Academic Medical Center, Amsterdam, The Netherlands

**Keywords:** Bezafibrate, Primary biliary cholangitis, Primary sclerosing cholangitis, Secondary sclerosing cholangitis, Pruritus, Itch

## Abstract

**Background:**

Pruritus (itch) is a frequent, burdensome and difficult-to-treat symptom in patients with cholestasis. Fibrates are currently under investigation for the treatment of primary biliary cholangitis in patients with a suboptimal response to ursodeoxycholic acid. Moreover, there is empirical evidence for a possible antipruritic effect. We aim to prove this in a randomized controlled trial, including patients with cholestatic liver diseases other than primary biliary cholangitis that are accompanied by pruritus.

**Methods:**

A multicenter investigator-initiated, double-blind, randomized placebo-controlled trial to evaluate the effect of bezafibrate on cholestatic pruritus in 84 adult patients with primary biliary cholangitis or primary/secondary sclerosing cholangitis. Primary outcome is the proportion of patients with a reduction of itch intensity of 50% or more (measured on a Visual Analog Scale) after 21 days of treatment with bezafibrate 400 mg qid or placebo. Secondary outcomes include the effect of bezafibrate on a five-dimensional itch score, liver disease-specific quality of life, serum liver tests and autotaxin activity. Safety will be evaluated through serum parameters for kidney function and rhabdomyolysis as well as precise recording of (serious) adverse events. We provide a schematic overview of the study protocol and describe the methods used to recruit and randomize patients, collect and handle data and perform statistical analyses.

**Discussion:**

Given its favorable safety profile and anticholestatic properties, bezafibrate may become the new first-line treatment option for treating cholestatic pruritus.

**Trial registration:**

Netherlands Trial Register, ID: NCT02701166. Registered on 2 March 2016;

Netherlands Trial Register, ID: NTR5436. Registered on 3 August 2015.

**Electronic supplementary material:**

The online version of this article (doi:10.1186/s13063-017-1966-8) contains supplementary material, which is available to authorized users.

## Background

A wide variety of cholestatic conditions are associated with pruritus (itch), including primary biliary cholangitis (PBC, formerly referred to as primary biliary cirrhosis [1]) and primary sclerosing cholangitis (PSC). The pruritus can occur locally or be generalized and is often reported by patients as the most burdensome symptom of their disease. Treatment options are limited as the pathophysiologic mechanism is largely unknown [2]. In our experience, resistant cases report severe sleep deprivation, depression and even suicidal ideations. In some patients, liver transplantation turns out to be the only option left [3].

### Fibrates in cholestatic liver diseases

Ursodeoxycholic acid (UDCA) is the only FDA- and EMA-approved therapy for PBC, improving transplantation-free survival. UDCA-responsive patients generally have similar life expectancy as sex- and age-matched controls [4]. Still, about 40% of patients do not respond to UDCA. Complementary treatment strategies are thus needed. In recent years, several case reports and pilot studies describe improvement of serum liver function tests upon fibrate treatment in patients with a suboptimal response to UDCA [5–20]. Whether or not fibrates improve transplantation-free survival in addition to UDCA remains to be proven in a currently ongoing phase III trial in PBC patients (www.clinicaltrials.gov: NCT01654731). Importantly, UDCA does not show benefit for pruritus in PBC and PSC. According to clinical observations [21], in line with some of the abovementioned reports [14, 17, 22, 23], bezafibrate does potentially have antipruritic properties. Anecdotally, itch complaints occur or recur after stopping the fibrates [21, 22].

### Bezafibrate as a potential alternative to current guideline-recommended antipruritic therapies

Rifampicin, the best available evidence-based treatment for the itch of cholestasis [4, 24, 25], has the disadvantage of hepatotoxicity in up to 12% [26–32] of patients with cholestasis during prolonged treatment. Moreover, rifampicin induces a wide variety of cytochrome P-450 (CYP) enzymes and, therefore, interacts with many different drugs. Fibrates would thus be an attractive alternative treatment for itch as they seem to be safe for long-term administration and seem to provide additional benefits to the course of the disease (at least, for PBC). Other treatment strategies are not as effective as rifampicin (e.g., bile salt sequestrants, naltrexone) or are experimental and much more invasive (e.g., nasobiliary drainage, plasmapheresis, ultraviolet-B phototherapy, liver transplantation) [4].

### Aim of this study: to evaluate the antipruritic effect of fibrates

The itch-relieving effect in the abovementioned studies was not measured as a primary outcome and was thus not systemically objectified by current quantification methods for itch intensity such as the widely used Visual Analog Scale (VAS) (validated in [33], discussed in [34]). Moreover, antipruritic effects were not controlled for while a considerable placebo effect should be taken into account (about 30% itch reduction was seen upon placebo treatment in one study [35]). Thus, we would like to validate the promising effect of fibrates on itch as a primary outcome in a double-blind, randomized placebo-controlled study.

### Proposed molecular antipruritic effect(s) of bezafibrate

As an agonist of peroxisome proliferator-activated receptors (PPARs), bezafibrate has anti-inflammatory [36–38], anticholestatic [20, 39–42] and antifibrotic [43] properties. In the current study we hope to identify its antipruritic mode of action. Our group recently showed that serum levels of lysophosphatidic acid (LPA) and serum autotaxin (ATX) activity, the enzyme forming the bulk of extracellular LPA, correlate with itch intensity in patients with cholestasis [44]. Our working hypothesis is that during cholestasis, increased serum ATX activity causes an increase in LPA-mediated activation of itch-specific sensory nerve endings. Importantly, successful guideline-approved and experimental antipruritic treatments (rifampicin, nasobiliary drainage and albumin dialysis) in patients with cholestasis correlated with a decline in serum ATX activity levels [45]. Thus, secondary objectives of the current study are determination of the effect of bezafibrate on serum ATX activity levels and/or screening for other pruritogens.

### Objectives

The primary objective is to determine the efficacy of bezafibrate in the treatment of moderate to severe cholestatic pruritus. Secondary objectives include efficacy in improvement of fatigue and liver disease-related quality of life and reduction of serum parameters of cholestasis, cholesterol, triglycerides and autotaxin activity. Safety will be evaluated through recording of (serious) adverse events ((S)AEs) and (suspected unexpected) adverse reactions ((SU)SARs) as well as serum parameters for kidney function and rhabdomyolysis. Also, the effect of stopping treatment on all the abovementioned variables will be assessed.

### Trial design

This is a multicenter, investigator-initiated, double-blind, randomized placebo-controlled trial.

## Methods

Methods are described according to the Standard Protocol Items: Recommendations for Interventional Trials (SPIRIT) 2013 Checklist for interventional trials which is provided in Additional file 2.

### Study setting

A total of nine academic hospitals are participating in this study, eight in the Netherlands and one in Spain (Barcelona). A list of study sites is provided at www.clinicaltrials.gov: NCT02701166.

### Inclusion criteria

Patients of 18 years or older with primary biliary cholangitis (PBC), primary sclerosing cholangitis (PSC) or secondary sclerosing cholangitis (as defined in the clinical practice guidelines of cholestasis from 2009 by the European Association for the Study of the Liver (EASL) [4]) can be included if they report an itch intensity of at least 5 out of 10 cm on a VAS twice within the week before inclusion with a minimum of 2 and a maximum of 7 days between both itch scores.

### Exclusion criteria

Subjects meeting any of the following criteria will be excluded from participation:

- Primary dermatologic abnormalities associated with pruritus
- Concomitant guideline-recommended as well as experimental antipruritic therapy, e.g., rifampicin, opioid-receptor antagonists (naltrexone, naloxone), serotonin-reuptake inhibitors (sertraline), ondansetron, phenobarbital, propofol, lidocaine, dronabinol, butorphanol, internal or external biliary drainage, extracorporeal albumin dialysis, ultraviolet-B phototherapy
  NB. Topical menthol-containing agents are allowed, as well as bile salt sequestrants (colesevelam, cholestyramin) as long as they are taken at least 4 h before or after intake of the study medication. Incidental use of these agents should be noted by patients in the diary, structural use should be noted on the Case Report Form (CRF)
- Pregnancy, women of childbearing potential not using contraception, breastfeeding
- Cholestasis due to obstruction that requires invasive desobstructive treatment within the time scope of the study (5 weeks) such as endoscopic retrograde cholangiopancreaticography (ERCP) or surgical removal of a tumor compressing the bile duct
- Use of opiates
- Renal insufficiency (creatinine clearance <60 mL/min)
  N.B. Concomitant use of UDCA is allowed

### Study interventions

Placebo tablets were developed matching the licenced Bezalip® Retard 400 mg Actavis tablets. The composition of the placebo tablets consists mainly of lactose monohydrate and excipients used were similar to the excipients of the uncoated Bezalip® Retard 400 mg Actavis tablets. Placebo tablets were manufactured under Good Medicinal Practice (GMP) licence.

**Fig. 1:**
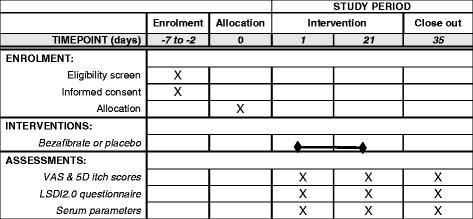
Standard Protocol Items: Recommendations for Interventional Trials (SPIRIT) figure: schedule of enrollment, interventions and assessments. *5D* five-dimensional, *LDSI* Liver Disease Symptom Index, *VAS* Visual Analog Scale

Additional file 3 provides a flow diagram of the study protocol. Additionally, a schematic overview according to the SPIRIT guidelines is provided in Fig. 1. Participation requires three 30-min outpatient clinic visits during the course of the study: at day 0 (start of treatment, bezafibrate or placebo), days 21 (end of treatment) and 35 (follow-up 2 weeks after stop of treatment).

### Criteria for withdrawal of study subjects

- Increase in serum transaminases (alanine aminotransferase (AST), alanine aminotransferase (ALT)) above six times the upper limit of normal *or* an increase of three or more times compared to the start of treatment
- If serum creatinine is within the normal range at the start of the study, an increase above 133 μmol/L is considered a reason for withdrawal. In case serum creatinine is increased already at start of the study we will allow an increase of 50% of serum creatinine concentration

### Procedures to monitor adherence to treatment

Tablets remaining after the treatment phase of the intervention will be used to assess adherence to treatment. Moreover, serum cholesterol and triglyceride levels will be tested, reflecting treatment adherence in the bezafibrate-treated group.

### Outcomes

#### Visual Analog Scale (VAS) itch score

The primary endpoint will be calculated from VAS scores obtained at study visits 1 and 2 (at start and stop of therapy), asking patients to report the maximum itch intensity of the past 24 h. We are using VAS itch scores as it is the most widely used method to assess itch intensity [33]. The scale consists of a 10-cm horizontal line divided into ten equal parts numbered 0 to 10. The 0 on the left is accompanied by a smiling face and the text “no itch at all,” the 10 on the right is accompanied by a sad-looking face and the text “worst itch possible.” Mostly to promote treatment fidelity, during the 21-day treatment period, patients will be asked to keep a diary to score their itch intensity (VAS) twice daily (after waking up in the morning and before going to bed in the evening) and make notes such as the use of co-medication, side effects and other information.

#### Five-dimensional (5D) Pruritus Scale

The 5D Pruritus Scale [46] is increasingly used in pruritus research, probably more accurately reflecting itch complaints as it evaluates multiple dimensions of itch during the course of the past 2 weeks: in addition to itch intensity (*degree,* range 1 (not present) to 5 (unbearable)), it assesses *duration* (range 1 (less than 6 h a day) to 5 (all day) and *direction* (range 1 (itch completely resolved) to 5 (getting worse). *Disability* is rated by the extent to which itch interferes with sleep (range 1 (never affects sleep) to 5 (delays falling asleep and frequently wakes me up at night) and social/leisure activities, homework/errands and professional activities (school/work) (all range 1 (never affects this activity) to 5 (always affects this activity)). *Distribution* is assessed by marking the affected skin surfaces on a ventral and dorsal body drawing which is an adaption made to the original version of the 5D Pruritus Scale where a list of body parts was listed in order to check affected sites. This way, we aim to calculate the extent of pruritic skin as a percentage of the whole body surface by the “rule of nine,” a method widely used to diagnose burn wound surface [47].

#### Liver Disease Symptom Index (LDSI) version 2.0

The LDSI2.0 [48] is a short questionnaire used to assess the potential effects of bezafibrate on other liver disease-related symptoms. It contains 18 questions regarding complaints of arthritis, pruritus, fatigue, abdominal pain, anorexia, jaundice and psychosocial consequences of the disease including depression and anxiety. Complaints can be rated on a 5-point scale; total score ranges from 18 to 90, higher scores reflecting worse symptoms.

#### Biological specimens

Serum will be collected from each participant at three time points: day 0, day 21 and day 35. Measurements of serum cholestasis parameters, including bilirubin, alkaline phosphatase (AP), gamma glutamyltransferase (γGT), ALT, AST, as well as albumin, lactate dehydrogenase (LDH), creatinine kinase (CK), low- and high-density lipoprotein (LDL, HDL) and total cholesterol, will be measured at the sites’ diagnostic laboratories. Furthermore, sample aliquots will be collected and stored at −80 °C until the last patient has completed the trial. Samples will be shipped on dry ice to the principal investigator’s laboratory.

Autotaxin (ATX) activity will be measured using an enzymatic endpoint assay, in which the amount of choline produced after 60 min of incubation of the sample with 1 μM of the substrate of ATX, lysophosphatidylcholine (LPC), is detected by fluorescence. Bile acid species will be determined using high-performance liquid chromatography (HPLC).

### Sample size calculation

We will compare the proportion of patients from both treatment arms who show a clinically relevant response to treatment which is defined as a reduction in itch intensity of at least 50%. Based on former placebo-controlled trials for cholestatic itch, selecting data only from patients with itch scores of 5 or higher at baseline who were treated for at least 2 weeks, we estimated that 18% of placebo-treated patients will show the defined response [30, 35, 49–51]. Based on empiric observations we believe that at least 50% of bezafibrate-treated patients will respond. Thus, we anticipate a difference in proportions between the bezafibrate and placebo groups of 32%.

Using Fisher’s exact test with alpha set at 0.05 we need 38 patients per group for a power of 0.80 for two-sided testing. Taking into account a 10% possible dropout we will include 84 patients (42 per arm) in this study. This power calculation was made after consulting a statistician and using the statistical program nQuery Advisor version 3.0.

### Recruitment

Patients are recruited from outpatient clinics of participating academic medical centers. After informed consent, based on oral and written information (see Additional file 1) provided by study physician or nurse, patients are enrolled at day 0 if the itch intensity is at least 5 out of 10 on a VAS, and all other inclusion and exclusion criteria are met. For all patients who drop out before day 21, we will include a replacement, whereas patients dropping out during the follow-up phase will not be substituted (see also Additional file 3).

The first participant was enrolled in April 2016. Recruitment is expected to be completed in 18 months, and will be enhanced by creating awareness among clinicians through regular presentation of the trial at (inter)departmental meetings. Moreover, we keep close contact with patient organizations to advertise the trial.

### Randomization and blinding

After informed consent, patients will be allocated randomly to either bezafibrate or placebo treatment (ratio 1:1) as per a computer-generated randomization schedule with randomly varying block sizes (maximum block size of 4) by the sites’ investigator through a web-based module written in ALEA (https://nl.tenalea.net, copyright NKI AVL, Amsterdam) by the AMC Clinical Research Unit. Stratification takes place for the VAS itch score at day 0 (2 strata: 5 cm ≤ VAS < 7.5 cm versus 7.5 cm ≤ VAS ≤ 10 cm). An automated email with the resulting randomization number will be sent to the trial pharmacy which allocates the patient to one of the two treatment arms and distributes the study medication accordingly to the patients, using identical packaging for placebo and bezafibrate tablets. This way, patients, physicians and outcome assessors stay blinded for treatment allocation. All investigators are aware that the pharmacy keeps the randomization list locked until all patients have completed the study, and that a request for unblinding can only be done in case a serious adverse event (SAE) occurs. Investigators at all sites keep their own subject identification log, in a secure place, and handle all personal data according to the local regulations on personal data protection.

### Data collection and management

Data collection will take place using electronic Case Report Forms (eCRFs, written by the coordinating investigator using web-based OpenClinica software (www.openclinica.com)) for the three visits, with built-in range checks for most variables. An appointed, independent monitor verifies entry of data on a regular basis by site visits according to a detailed monitoring plan.

From the data collection tool, data can be exported directly to the database software (SPSS). The coordinating investigators manage coded data by regular backups on a secure drive. Site agreements have been signed by coordinating and participating centers to assure agreement about access to, and use of, trial data.

### Statistical analysis plan

Statistical analyses will be performed using SPSS (version 22 or above) according to intention-to-treat principle: patients who dropped out before the end of treatment because of lack of treatment efficacy will be included in sensitivity analyses. The nature and extent of any missing data and coding/typing errors will be addressed in a blinded fashion and dealt with accordingly following general principles (e.g., imputation and/or the use of specific statistical models), as will be described in the resulting manuscript.

Statistical tests will be two-tailed. *P* values < 0.05 will be considered as statistically significant. Baseline characteristics of the study population in both groups will be depicted in a table, summarizing means ± standard deviations of continuous variables and frequencies and proportions (%) for categorical variables. The primary outcome measure will be depicted in a graph. Specific statistical methods to analyze the most important outcome measures are addressed below. Results of these analyses (% change in each group, *P* values) will be presented in a table.

#### Primary outcome

The effect of bezafibrate on itch will be confirmed by a Fisher’s exact test on the difference in the proportion of patients responding to treatment (≥50% reduction of itch intensity in the VAS score at day 21 compared to day 0) between the bezafibrate and the placebo groups.

#### Secondary outcomes

In exploratory analyses, we will test:

- The absolute effect of a 3-week treatment as well as a 2-week follow-up on VAS and 5D Pruritus Scale scores (day 0 versus day 21 and day 21 versus day 35) as well as the difference of these effects between both groups (unpaired *t* tests)
- The effect of a 3-week treatment on daily morning and evening VAS scores in both groups separately (repeated measures analysis of variance (ANOVA) days 1–21)
- The difference in time at which patients reach the primary endpoint (50% reduction in itch intensity) for the first time between both groups (unpaired *t* test)
- The difference in morning and evening VAS scores within patients at day 1 (irrespective of treatment group, paired *t* test)
- The difference in the effect of a 3-week treatment as well as a 2-week follow-up on LDSI2.0 questionnaire total and subdomain scores between both groups (day 0 versus day 21 and day 21 versus day 35, unpaired *t* test)
- The difference in the effect of a 3-week treatment as well as a 2-week follow-up on serum ATX activity, AST, ALT, ALP, γGT, bilirubin, albumin, creatinine, CK, LDH, glucose, total cholesterol, HDL- and LDL-cholesterol and triglycerides between both groups (day 0 versus day 21 and day 21 versus day 35). Depending on the distribution of the data, parametric or nonparametric tests will be chosen, i.e., unpaired *t* test and Mann-Whitney *U* test, respectively
- The number of AEs and SAEs during and after treatment will be listed in a table. As few AEs are expected, no statistical analysis will be applicable to compare between groups

#### Associative analyses

Univariable and, if applicable, multivariable analysis will be performed to find out if prerandomization itch intensity stratum, gender, age, Body Mass Index (BMI), underlying liver disease, disease stage and/or use of co-medication relate to treatment outcome.

#### Interim analysis

As we are not studying an intervention for life-threatening disease, no interim analysis will be performed to be able to keep investigators blinded until all 84 patients have completed the study.

## Discussion

The treatment of cholestatic pruritus is one of the major challenges in the daily practice of hepatologists. This, together with the promising effect of empirical bezafibrate treatment, was reason for the NASL Cholestatic Liver Diseases Study Group in collaboration with the University of Barcelona to initiate this placebo-controlled trial.

Although rifampicin is available [4], there is considerable resistance among clinicians (and patients) to prescribe (or take) rifampicin due to the risk of hepatotoxicity [26–32]. Bezafibrate would be an attractive alternative given its favorable side-effect profile and disease-modifying properties (in PBC). We choose to first show the efficacy of bezafibrate in a placebo-controlled trial before we consider continuing by showing noninferiority to rifampicin which would require many more participants. A 3-week treatment period seemed appropriate to us, as empirical observations found that this was long enough to perceive an antipruritic effect while we consider that this period of time is still ethically acceptable for patients receiving placebo, as most participants have likely suffered from itch for months or years before participation to the trial (due to, in our experience, patients’ and/or doctors’ delay and prior trial and error of antipruritic therapies). Because of this relatively short treatment duration, participants are requested not to use any rescue medication during the course of the study other than use of topical agents (e.g., menthol cream) and bile sequestrants (e.g., colestyramin), of the use of which should be noted in the diary.

It should be noted that the manufacturer of bezafibrate states that it is contraindicated in patients with liver disease. We believe, however, that with about 20 pilot studies [4–20, 22, 23] performed in patients with PBC and PSC, having increased AP levels as an inclusion criteria and all showing stable or decreasing liver transaminase levels during long-term treatment, it is safe to administer bezafibrate for 3 weeks in our target population.

We choose to only include patients with an itch intensity score of 5 cm or higher on a 10-cm VAS in order to make the primary endpoint, a reduction of itch intensity of 50% or more, clinically relevant. We believe the VAS score is the best available but still a subjective measure, and the cutoff point of 5 cm has been chosen somewhat randomly. To prevent bias we cannot reveal this inclusion criterion to patients. This lack of transparency may cause some indignation, however, especially in those who report an itch intensity score of just below 5 cm and have persistent itch despite having tried all currently available options. Also, patients who experienced relief of their complaints by taking the study medication may be reluctant to stop treatment after 21 days. For these groups of patients we can only advise clinicians to consider prescribing bezafibrate off label, pending the trial’s results.

Given the marked placebo effect on pruritus intensity observed in previous clinical trials, as described above, we have made maximum effort to assure blinding of patients, physicians and study personnel involved. The use of web-based randomization module and subsequent distribution of study medication by a central pharmacy (one in The Netherlands and one in Barcelona) directly to the patients minimizes the risks of unblinding. Patients and their physicians may suspect the nature of study medication administered if, after completing the trial, bezafibrate is prescribed off label, depending on the response to that treatment. We believe, however, this will not affect the reliability of the data yet collected or processes of recruitment and data collection by the involved physician.

Although we are capturing itch intensity data using various measures in parallel (VAS, 5D and LDSI2.0 itch scores during the three visits as well as daily morning and evening VAS scores from the diaries), we endeavor to avoid the statistical multiplicity problem by having defined a single confirmatory primary endpoint, based on VAS scores during the study visits. All other data will be used in exploratory analyses only, as defined in the protocol. For example, previous studies described diurnal variation of itch intensity [2, 52] where most patients reported more intense itch in the late evening and early nighttime. By using diaries, we will also acquire information on how fast a possible effect occurs. In addition, we hope that the use of a diary stimulates compliance and increases accuracy of co-medication usage and reports of side effects.

### Trial status

Recruitment of patients started in March 2016 and is ongoing at the time of submission of this manuscript.

## Additional files

Additional file 1: Informed consent materials: Patient Information Letter and Informed Consent Form. (PDF 1429 kb)

Additional file 2: SPIRIT Checklist: overview of items recommended by the SPIRIT guidelines, addressed throughout the manuscript. (DOC 125 kb)

Additional file 3: Flow diagram of the FITCH study protocol. Abbreviations: *5D* five-dimensional, *LDSI* Liver Disease Symptom Index, *VAS* Visual Analogue Scale. (PDF 49 kb)

## Abbreviations

ALT: Alanine aminotransferase
AMC: Academic Medical Center
ANOVA: Analysis of variance
AP: Alkaline phosphatase
AST: Aspartate aminotransferase
ATX: Autotaxin
BMI: Body Mass Index
CK: Creatinine kinase
CYP: Cytochrome P-450
ERCP: Endoscopic retrograde cholangiopancreaticography
GCP: Good Clinical Practice
GMP: Good Medicinal Practice
HDL: High-density lipoprotein
LDH: Lactate dehydrogenase
LDL: Low-density lipoprotein
LDSI: Liver Disease Symptom Index
LPA: Lysophosphatidic acid (lysophosphatidate)
MREC: Medical Research Ethics Committee
NSAIDs: Nonsteroidal anti-inflammatory drugs
PBC: Primary biliary cholangitis (formerly primary biliary cirrhosis)
PPAR: Peroxisome proliferator-activated receptor
PSC: Primary sclerosing cholangitis
SAE: Serious adverse event
UDCA: Ursodeoxycholic acid
VAS: Visual Analog Scale
γGT: Gamma glutamyltransferase
