# Supplementary material for: Fibrates for the treatment of cholestatic itch (FITCH): study protocol for a randomized controlled trial
Source: Trials. 2017 May 23;18:230. doi: 10.1186/s13063-017-1966-8 (PMC5442649; doi:10.1186/s13063-017-1966-8)
Supplement: Supplementary file 1 — Informed consent materials: Patient Information Letter and Informed Consent Form. (PDF 1429 kb) [file 13063_2017_1966_MOESM1_ESM.pdf]

FITCH trial: the effect of bezafibrate on itch in liver diseases.

Dear madam/sir,

Hereby we would like to ask you to participate in a medical research project investigating a new treatment for itch with liver diseases, named the FITCH trial (composed of the F of fibrate and itch, the symptom we would like to find a new treatment for). You can decide to participate or not. Before you make the decision, it is important that you know some more details about the trial. Please read this information letter carefully. Discuss it with your partner and/or relatives. You can also always ask an independent physician, who knows about the trial, for assistance in your decision to participate or not.

May you have remaining questions after reading this letter, please do not hesitate to contact the investigator (for contact information see appendix 4).

1. What is the aim of this trial?

Some patients with a liver disease suffer from itch. The itch can be so intense that it greatly affects patients' quality of life. As the cause of this itch is not known, there are not many effective medicines available. The aim of this study is to test the drug bezafibrate for it's effect on itch to be able to make it available as a new treatment for these patients. Also, we hope to gain knowledge about the cause of itch in liver diseases.

2. Which drug is investigated?

We investigate the effect of bezafibrate on itch in liver diseases. Bezafibrate is a drug that is prescribed before to some patients with primary biliary cirrhosis (PBC). To be able to reliably determine the effect, half of the patients will receive bezafibrate and half of the patients will receive placebo. The placebo is a fake drug that looks exactly like bezafibrate but does not contain any active substances.

3. How do we investigate this?

In the week before start of the treatment we ask you to twice score your itch intensity on a line from 0 to 10. The study nurse or physician will judge your eligibility to participate based on fixed criteria. A computer then assigns you randomly to one of the two treatment groups (bezafibrate or placebo). This is a double-blind trial; meaning that neither the patient nor the doctor knows which group the patient is assigned to. The treatment has a duration of 3 weeks. In total, the trial takes 5 weeks. In the appendix you'll find a timeline explaining the activities in detail.

4. What do we expect from you?

Take the study medicine once a day during a meal. We will give you a diary in which we ask you to keep track of your itch intensity twice a day. You will have to come to the hospital three times.
It is important to not take other anti-itch medicine during your participation in this trial, except for menthol cream. Whenever you use this cream, please make a note of this in the diary.
There is no restriction for other medicines except for strong painkillers (opiates). Lighter painkillers, such as paracetamol (acetaminophen) and ibuprofen or diclophenac are allowed. If you are in doubt please discuss this with your physician or the investigator (appendix 4).
However there are no data about a potential harmful effect of the study medication on pregnancy, we advise you to not get pregnant during the 5 weeks of the trial. Contraceptives can be

discussed with your physician. If you may get pregnant during the trial please contact your physician as soon as possible.

5. What is different from a normal treatment?
Participation with this study comprises a 50% chance that you will receive a placebo. You cannot influence the chance of receiving the study medication.
On the first and last day of the treatment and 2 weeks after stop of the treatment an extra vial of blood will be withdrawn (in addition to blood withdrawn for investigations by your own doctor). On those days we also ask you to fill in 2 questionnaires.

6. What are the alternative treatment options?
These differ from patient to patient and should be discussed with your own physician.

7. What side effects can you expect?
The medication used in this trial may have the following side effects:

- loss of appetite
- nausea, stomach pain, diarrhea, bloating
- fatigue, somnolence, headache, dizziness
- depression
- skin rash
- dyspnoea, shortness of breath
- possible influence on sperm cells and thereby the fetus

Some side effects are very rare. You can read more about this in appendix 3.

8. What are the possible (dis)advantages of participation to this trial?
An advantage of participation may be that bezafibrate can soon be prescribed to you and other patients with itch complaints. If bezafibrate turns out to be effective against itch, it represents a very attractive alternative to the current treatment options.

The most important disadvantages are the change that you will receive a placebo for 3 weeks and the chance that bezafibrate is not effective against itch. In that case, you may suffer from itch longer than if you would not have participated. Furthermore, participation will cost some of your time, as we ask you to come to the hospital 3 times during the 5 weeks of the trial.

9. What happens if you do not want to participate?
You can decide for yourself to participate or not. Participation is voluntary. If you decide not to participate you don't have to take further action. It is not necessary to give a reason to not participate. You will receive the treatment you would also get if you were not asked to participate. If you decide to participate now, you may at any time during the trial still decide to quit. There is no risk in stopping early.

11. What happens after the trial?
After the 5 weeks of the trial, or if you have decided to withdraw before the end of the trial, you will still be unaware of which treatment (bezafibrate of placebo) you have used. Only after all 84 participants have completed the study, you and your doctor will be informed about what medicine you have received. Only in exceptional situations, such as emergencies, your doctor may ask the AMC pharmacy to disclose the treatment.

There are no risks attached to withdrawal from the trial at any point. Your physician will discuss with you the possibilities for further treatment of your itch complaints.

12. Is there insurance for participants?
There is insurance for all participants. The insurance covers all damage that may be caused as a consequence of your participation in this trial. In the appendix you will find more information.

13. Will you be informed about any changes in the trial protocol?
The trial will be executed as much as possible according to the protocol. However, things may change. For example, if you or another patient report unexpected or severe side effects or if new information becomes available. If so, you will be informed. You may then always revise your decision to participate. If we believe you are in danger we will stop the trial.

14. What happens with your personal details?
Your personal details will be treated confidentially. Data from each participant will be encoded, and only the principle investigator has access to the key of these codes. Also in any audits, information will stay encoded. Your name and address will be disclosed to the AMC trial pharmacy, in order to send the medication to you. Participation with this study implies you give permission to do so. Your name and address will not be used for other purposes than to send you the medication.
Some people have permission to break the code. This may be necessary to check the integrity of the trial. People who are allowed to break the code are the investigator, the doctor or research nurse that has asked you to participate, an independent auditor or the governmental inspection. They will all treat your personal data with confidentiality.
We are obliged to save all research data for 15 years. You give permission for this when you decide to participate. If you disagree with this, you cannot participate.

15. Will your general physician be informed?
We will inform your general physician about your participation in this trial. This is for your own safety. You give permission for this when you decide to participate. If you disagree with this, you cannot participate.

16. Are there extra costs and/or is there a financial compensation for participation with this trial?
You will have to come to the outpatient clinic 3 times during 5 weeks to fill in the questionnaires and withdraw blood. We will provide compensation for travel expenses made for these visits. Please use the provided declaration form and hand it over to your study physician or nurse, or send it to the coordinating investigator (see appendix 4 for contact information).

17. Who has approved this study?
The ethics committee of the Academic Medical Center (AMC) in Amsterdam has approved this study. For more information, please read appendix 1.

18. Is there anything else you want to know?
In case of further questions please inform the investigator or the assigned independent expert in your hospital.

19. Appendices

1. General brochure about participation in medical research
2. Trial scheme
3. Side effects of study medication
4. Contact information involved physicians and investigators
5. Insurance information

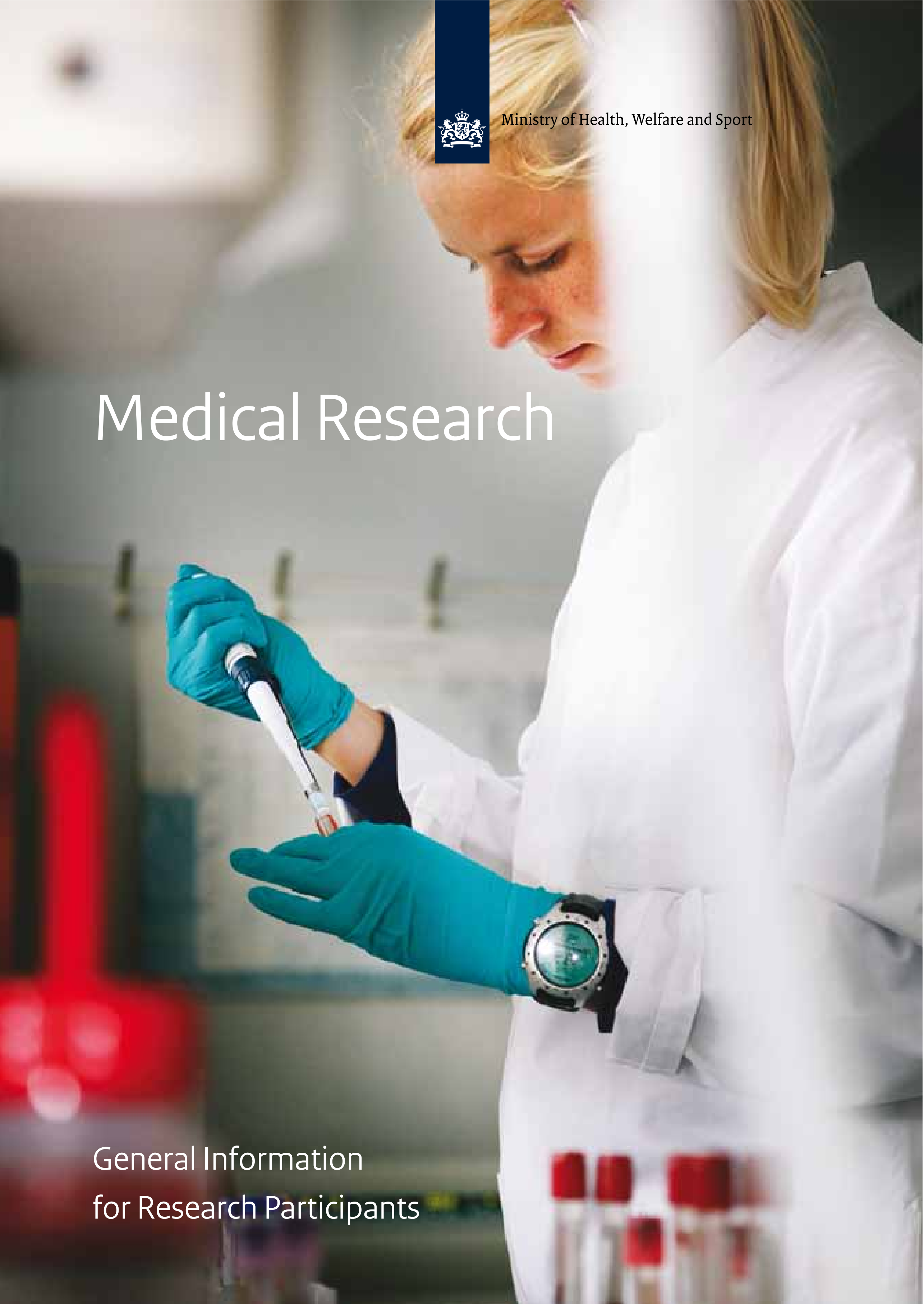

Ministry of Health, Welfare and Sport

Medical Research

General Information for Research Participants

Contents

Introduction 5

Medical research 6
- What is a medical research?
- Who is classified as a research participant?
- Who conducts the research?
- What does the research involve?

Participation 8
- Who decides whether you participate?
- What are the benefits for you?
- What do you need to take into account?
- What is the procedure for participation?
- What is the procedure if you do not wish to participate?

Rights and obligations 11
- What are your rights as a research participant?
- What are your obligations as a research participant?

Monitoring 13
- Who monitors the progress of the research?
- What happens if something goes wrong?
- What happens to your data?

More information 14
- Where can I find more information?
- Where do you complain?

Annex 1: Questionnaire 16

Annex 2: The development of new medications 18

Annex 3: Space for own questions and notes 19

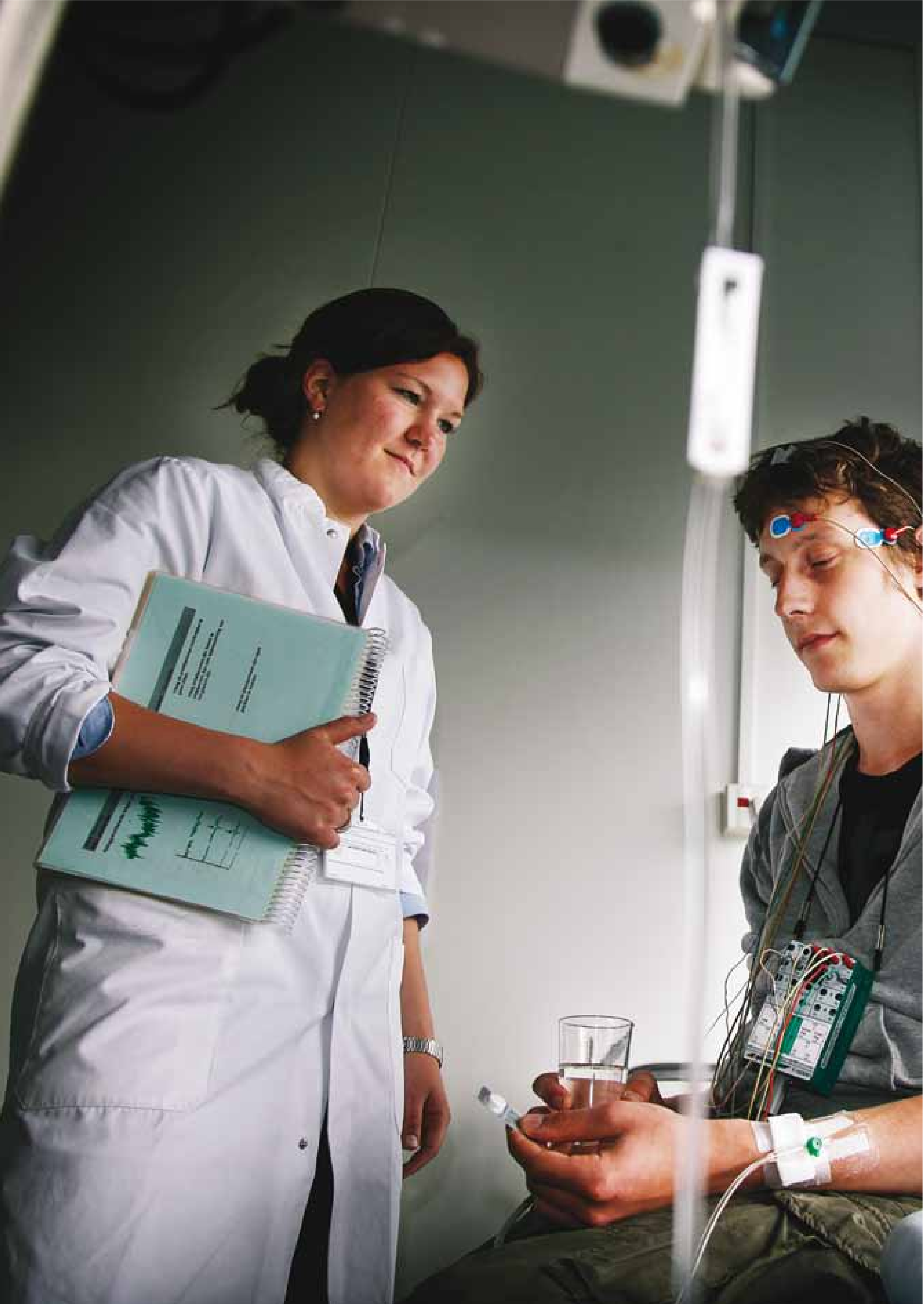

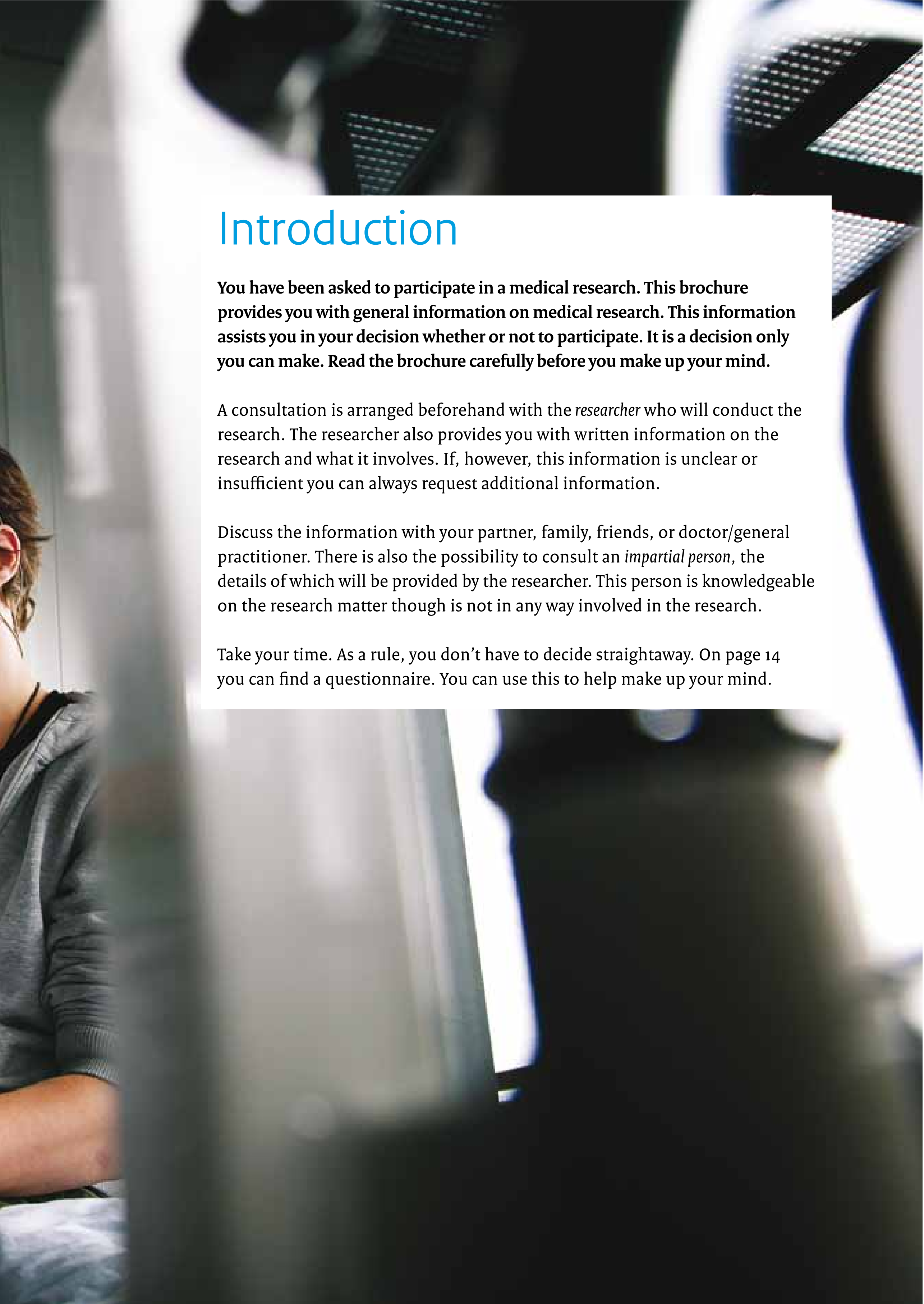

Introduction

You have been asked to participate in a medical research. This brochure provides you with general information on medical research. This information assists you in your decision whether or not to participate. It is a decision only you can make. Read the brochure carefully before you make up your mind.

A consultation is arranged beforehand with the *researcher* who will conduct the research. The researcher also provides you with written information on the research and what it involves. If, however, this information is unclear or insufficient you can always request additional information.

Discuss the information with your partner, family, friends, or doctor/general practitioner. There is also the possibility to consult an *impartial person*, the details of which will be provided by the researcher. This person is knowledgeable on the research matter though is not in any way involved in the research.

Take your time. As a rule, you don't have to decide straightaway. On page 14 you can find a questionnaire. You can use this to help make up your mind.

Medical research

What is a medical research?

There are two types of research. If you suffer from physical complaints you go to the hospital for research or examinations. A doctor examines you to determine the cause of your complaints. The purpose is to make you better.
This is called *diagnostic research or examinations*. There is also *medical research*.
There are three types of medical research:

1. Research for improving treatments for illnesses
Examples: A more effective medication for headaches.
A new type of heart valve.

2. Research for finding out more about illnesses
Examples: What causes ectopic pregnancy?
Can you get high blood pressure from eating liquorice?

3. Research aimed at detecting illnesses
Example: How can we detect breast cancer at an early stage?

In other words, the primary purpose of medical research is not to cure you of an illness or ailment. In this brochure you will find out more on medical research.

Who is classified as a research participant?

Anyone who participates in a medical research is a *research participant*. There are two types of research participants: healthy volunteers and patients. Patients may benefit from taking part in the research.

Who conducts the research?

The research is conducted by a researcher. This person is a qualified doctor or scientist and has a great deal of knowledge on the research field.

What does the research involve?

During the research a new treatment, operation of medication may be tested.
This will be hereinafter referred to as '*treatment*'.

The researcher usually compares a new treatment with an existing one. The receivers of both treatments are chosen at random from the group of participants. This process is called *randomisation*. It is matter of chance to which group you are assigned.

The researcher is often unaware of the group to which you are assigned. The research is then termed *double blind*. This ensures a more neutral manner of comparing the results of both groups. If deemed necessary, the researcher can find out to which group you were assigned.

The researcher may compare a new treatment with a fake one. This fake treatment is called a *placebo*. The placebo appears identical to the new treatment. One group of research participants receives the new treatment while the other receives the placebo.

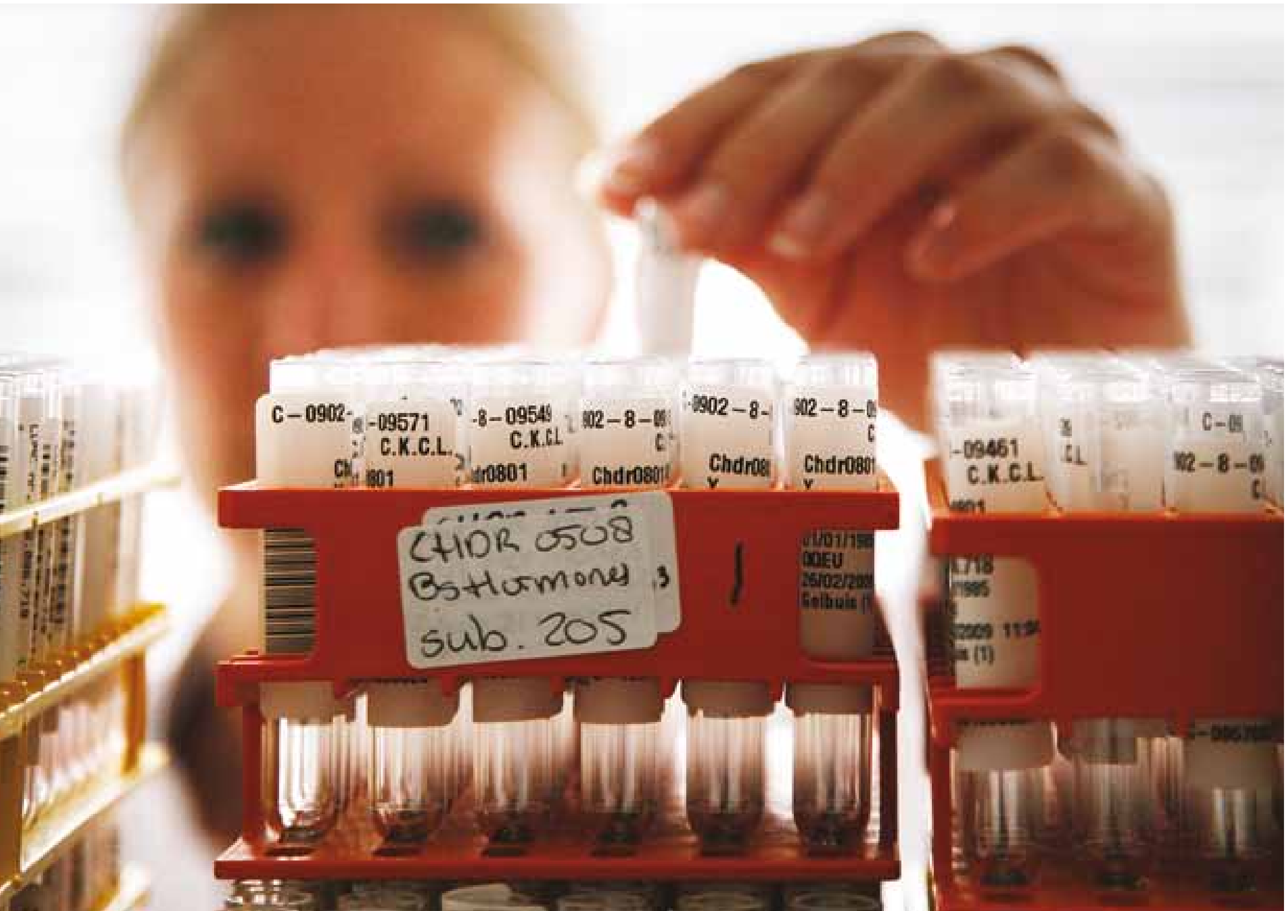

Participation

Who decides whether you participate?
It is entirely up to you whether you participate in a research. It is *voluntary*. You are never under any obligation to participate. Only do so if you are sure you are fully aware of the contents, possible risks and benefits of the research.

What are the benefits for you??
- You make a contribution to medical progress.
- Are you a patient? If so, you may benefit from a new treatment. But then again maybe not. The researcher can tell you more on this. Participation is on a voluntary basis and is therefore unpaid. Travel expenses are usually reimbursed.
- Are you a healthy volunteer? Then you may receive financial compensation. Your travel expenses are always reimbursed.

What do you need to take into account?
- Are you a patient? If you participate you usually have to return several times for checkups. So participating involves time and effort.
- Are you a healthy volunteer? Then participating costs time. Perhaps half a day or several short visits. You may even be required to spend one or two days in a clinic.
- Participating is not without its risks. As the treatment is new and still being studied, not all possible effects and side effects are known. The researcher often also conducts extra tests or takes blood samples. How great the risk is depends on the type of research and your state of health. The researcher explains this to you.
- Participating may be stressful or unpleasant as:
  - extra physical or internal examination may be necessary;
  - you may be asked questions about distressing experiences;
  - you may be required to stop taking medication which you normally take;
  - the research may involve special rules, for example on the use of contraception.

What is the procedure for participation?
If you wish to participate, you are required to sign a declaration. This is called a declaration of consent (toestemmingsverklaring). By signing you state that you are participating voluntarily. You receive a copy of the signed declaration.

Your signature does not mean that you must complete participation in the research. You always hold the right to refuse participation or to withdraw from the research programme at any time.

You often first undergo a physical examination, in which the researcher will determine if you are physically fit for the research. A possible outcome is that you are deemed unfit to participate due to, for example, high blood pressure.

What is the procedure if you do not wish to participate?

If you decide not to participate you are not required to do or sign anything. You do not need to explain why you do not wish to participate. Are you a patient? Then you simply receive the treatment that you would otherwise normally receive.

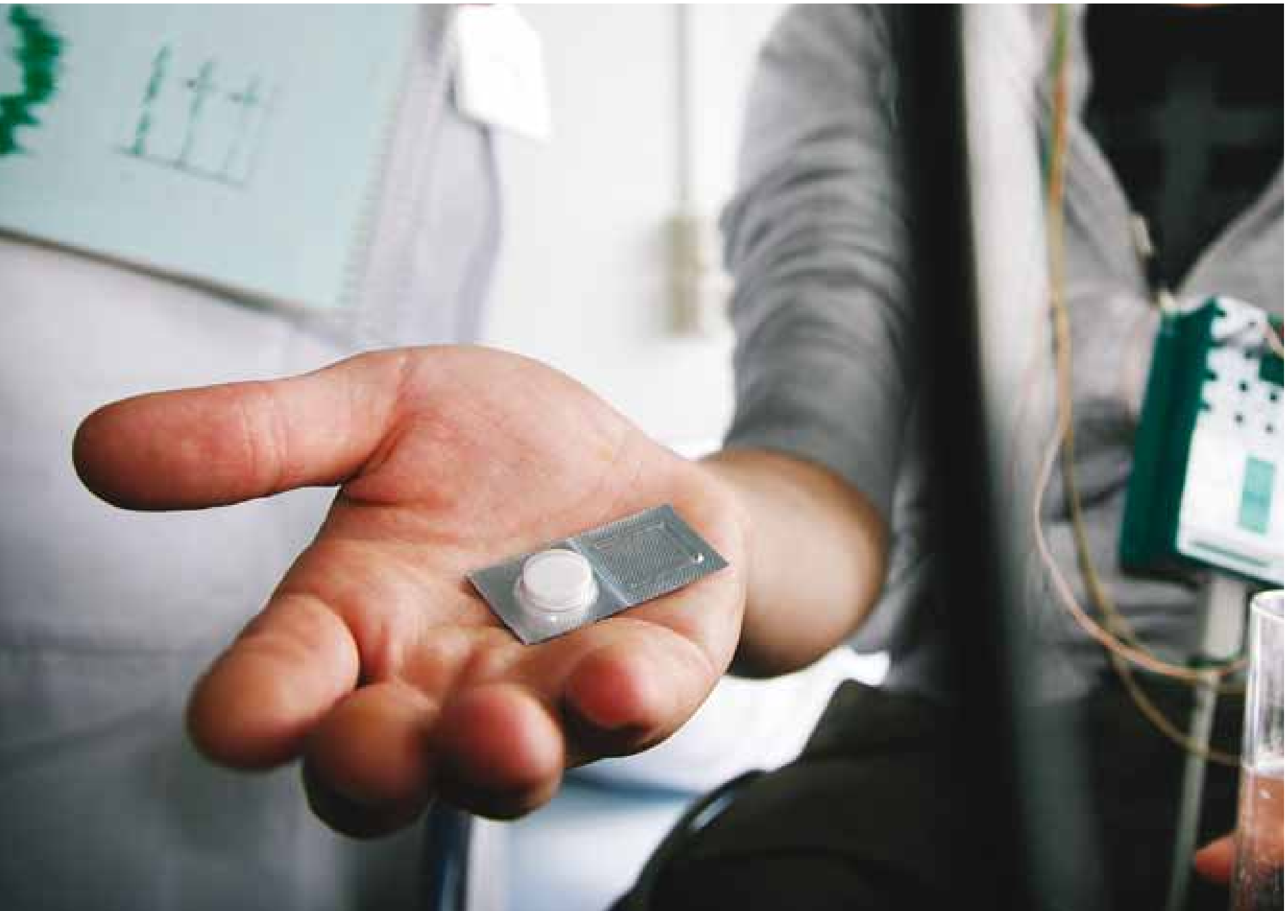

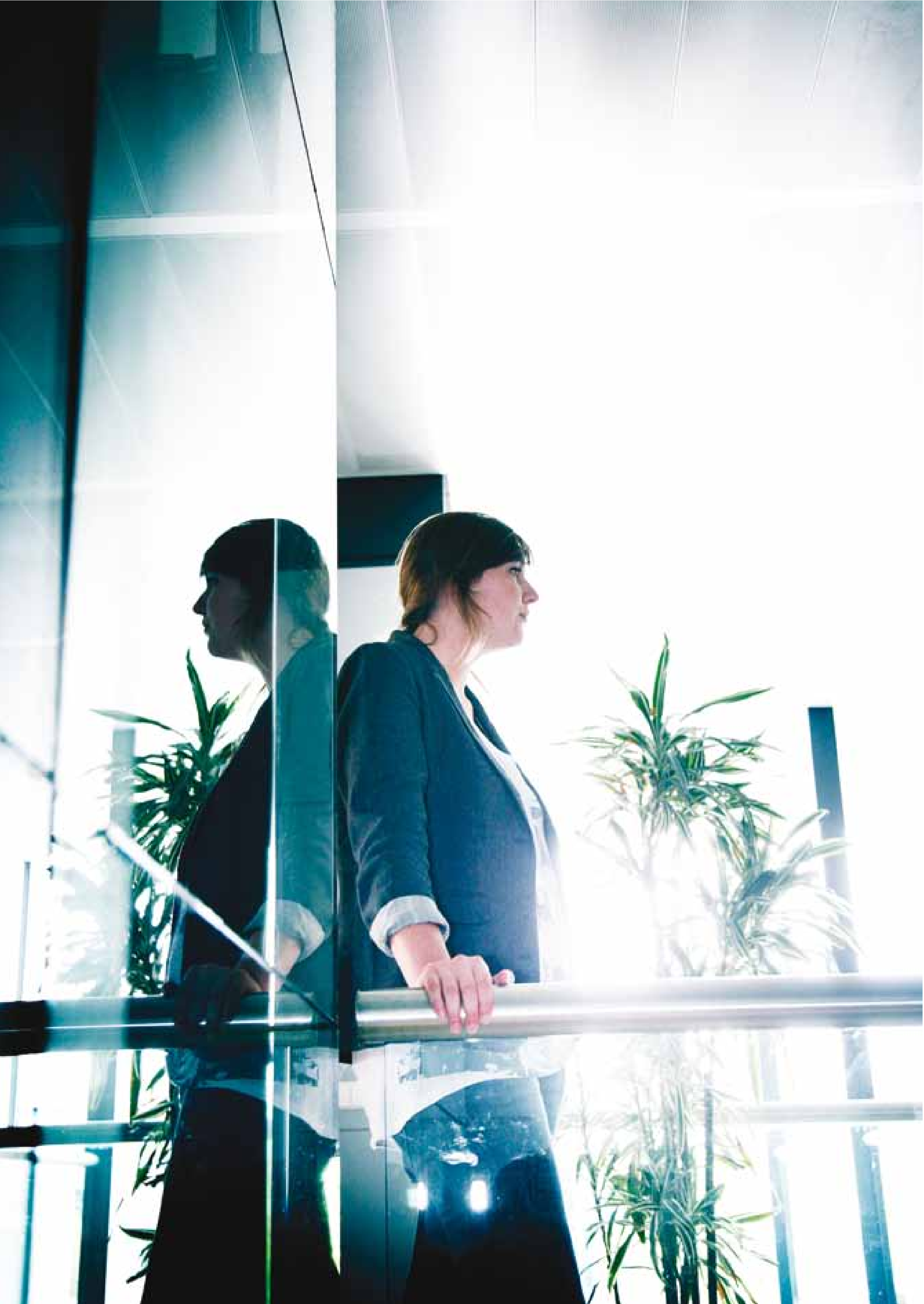

Rights and obligations

What are your rights as a research participant?
You have rights as a research participant. These are laid down in law.
The main ones are:

The right to make your own decision
You decide yourself whether to participate in a medical research. It is up to you. Even if your doctor asks you to participate you can always refuse.

The right to be informed and ask questions
The researcher is obliged to meet you first for a consultation. He/she is also obliged to provide you with written information on the research. You can ask any questions you may have. You can do this before, during and after the research. The researcher is obliged to answer your questions.
You will be provided with details of an *impartial person*. This person is knowledgeable on the subject, but is not involved in the research itself. You can also ask this person questions about the research.

The right to time to think
You are not usually required to make an immediate decision whether to participate in a research. You have the right to read the information at your own pace and in the comfort of your own home. There are however instances when a quick decision is required, for example in cases of emergency medical assistance.

The right to leave the research at any time
You may at any point express your wish not to participate. Even if the research has already started. You don't have to give a reason for your wish to leave the research. If you leave while the research is underway it will not influence the treatment you were receiving before the research began.

There may be times when immediate departure from the research programme is not possible due to possible health risks as a result of doing so. So if you do wish to leave always inform the researcher beforehand.

The right to protection of your data
During the research the researcher will gather data on you. This data is treated as confidential. Your data is given a code and your name is omitted. Your name will not be mentioned in any research reports. For more information, see 'What happens to your data?' on page 10 of this brochure.

What are your obligations as a research participant?

You must adhere to the rules of the research. These rules differ per research. You may have to start the research on an empty stomach. This means not eating after the evening before the research begins. You may only drink water. You may have to take a pill at the same time every day.

It is important that you adhere to the rules. Otherwise, the researcher is unable to conduct the research properly and the results will be unreliable. The researcher may then decide that you can no longer participate in the research.

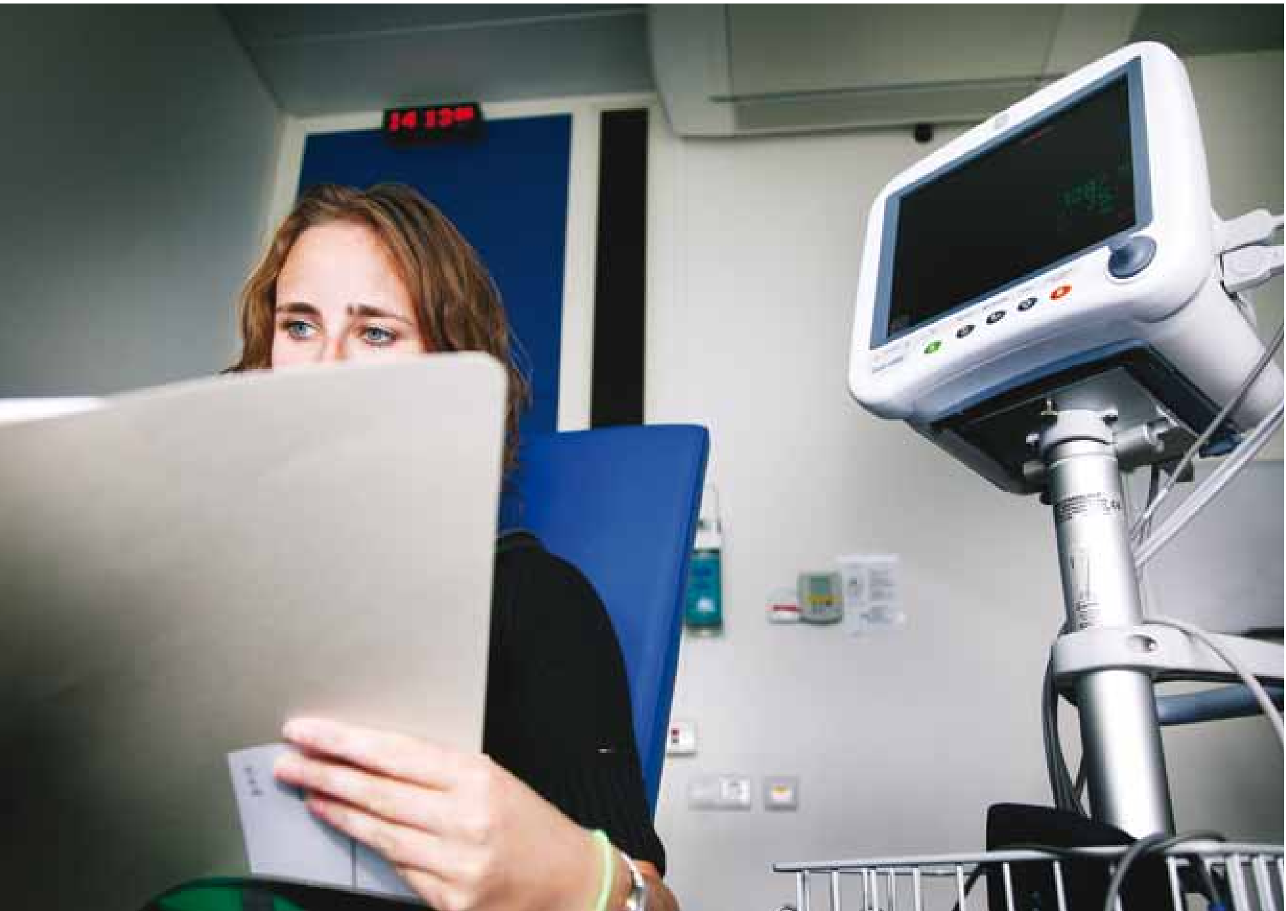

Monitoring

Who monitors the progress of the research?

The Netherlands has stringent rules for the conducting of research with human participants. These rules are laid down in the *Medical Research (Human Subjects) Act*.
An appointed committee reviews each research in advance. This committee is called the *review committee*. A research may only take place after approval has been given by the appointed review committee.
The review committee reviews amongst other things:

- whether the research is correctly set up;
- whether the information you receive is correct;
- whether the risks involved in the research are too great;
- whether the burden for the research participant is too high.

The review committee's members are experts on the field of research. They are, for example, doctors, or are very familiar with the laws and regulations in this area. They have no personal gains or interest in the research.
You will find details on the ethics committee reviewing the research in the information provided by the researcher.

What happens if something goes wrong?

It is the researcher's responsibility to prevent anything going wrong in a research. Despite this it is possible that problems occur. Which is why research participants are insured in the event anything does happen. If you suffer any complaints as a result of the research you will be compensated by the insurance company.
The researcher will provide you with information on the insurance policy.

What happens to your data?

Any personal data gathered by the researcher during the course of the research remains confidential. The researcher stores your data under a code. This code is used in any reports on the research. Only the researcher knows the code given to your data.

Reliability

One or two other people may see your data. These people check whether the research is sound and reliable. This procedure is laid down in the *Personal Data Protection Act*.

People who have access to your data are, for example:
• the research team;
• the manufacturer of the treatment being researched;
• the review committee;
• the safety committee that monitors the research;
• the Health Care Inspectorate.

During the research
The researcher will hold on to your data during the research. He/she tells you how the data will be used. You only give permission for the use of your data for this particular research. Your data has to be kept for a while after the research has been completed. After this it will be destroyed.

Further research
You can give permission for the use of your data for further research. The researcher will then keep your data. If your data is used for another research or for any other purpose the researcher will ask you again for permission.

More information

Where can I find more information?
• Ask the researcher. He/she will supply you with written information. This information is specific to the research you have been asked to participate in. Do not hesitate to ask the researcher any further questions.
• Ask the impartial person. You can find this person's details in the written information you receive from the researcher. Are you a hospital patient? You can often contact the patient service department.
• Go to www.ccmo.nl. This is the website of the Central Committee of Research involving Human Subjects (CCMO). The CCMO closely monitors medical research involving human participants. On this website you can find more general information. You can also read which regulations researchers have to adhere to. On the website click on the link 'voor publiek' (Dutch only).

Where can I complain?
If you have a complaint you can first discuss with the researcher. If you prefer not to, you can contact the complaints committee of the institute where the research is being conducted. You can find the phone number in the written information provided by the researcher.

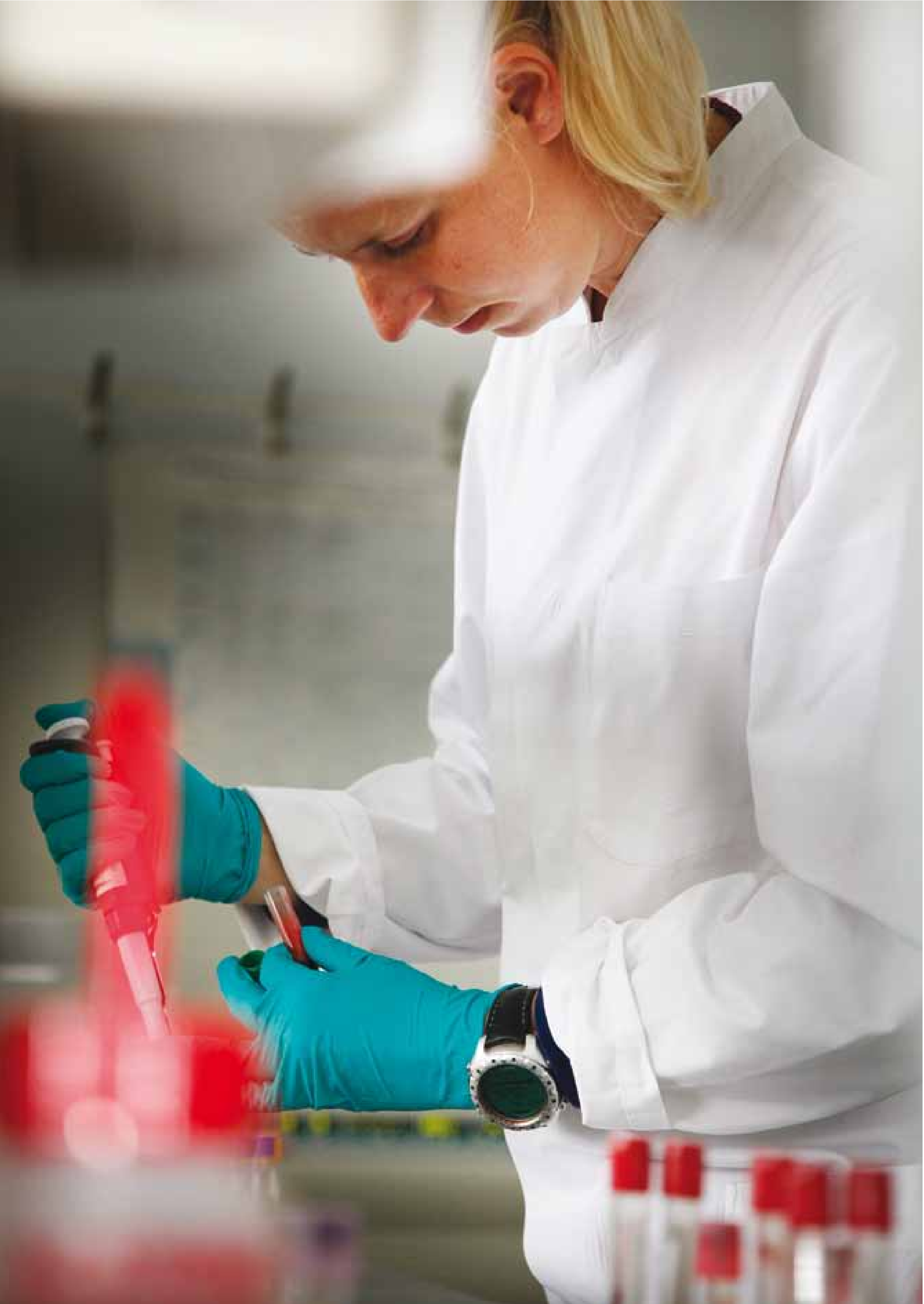

Annex 1: Questionnaire

Read these questions before you decide whether to participate. The questions may help you make up your mind. You can find the answers in this brochure and in the written information on the research itself. The researcher will provide you with this information.

1. What is the purpose of the research?
2. (How) will I benefit from the research?
3. Why has the researcher asked me to participate?
4. How much time will the research take?
5. What exactly do I have to do as a research participant?
6. What are the possible risks or side effects?
7. I am trying to get pregnant. Can I still participate?
8. Do I have to stop taking my own medication?
9. What happens if something goes wrong during the research? Who can I consult?
10. How am I insured? What does the insurance policy cover?
11. What happens to my data?
12. Am I allowed to find out my own results?
13. When will I find out which treatment I was given?
14. When will the results of the research be made known? Will I receive a copy?
15. What happens once the research is completed? Can I continue to take the studied treatment if it was effective in my case?
16. Who can I contact if I have any more questions?

Tip
You can take someone along with you to your consultation with the researcher. Two pairs of ears can be better than one. It may also help to write important points down.

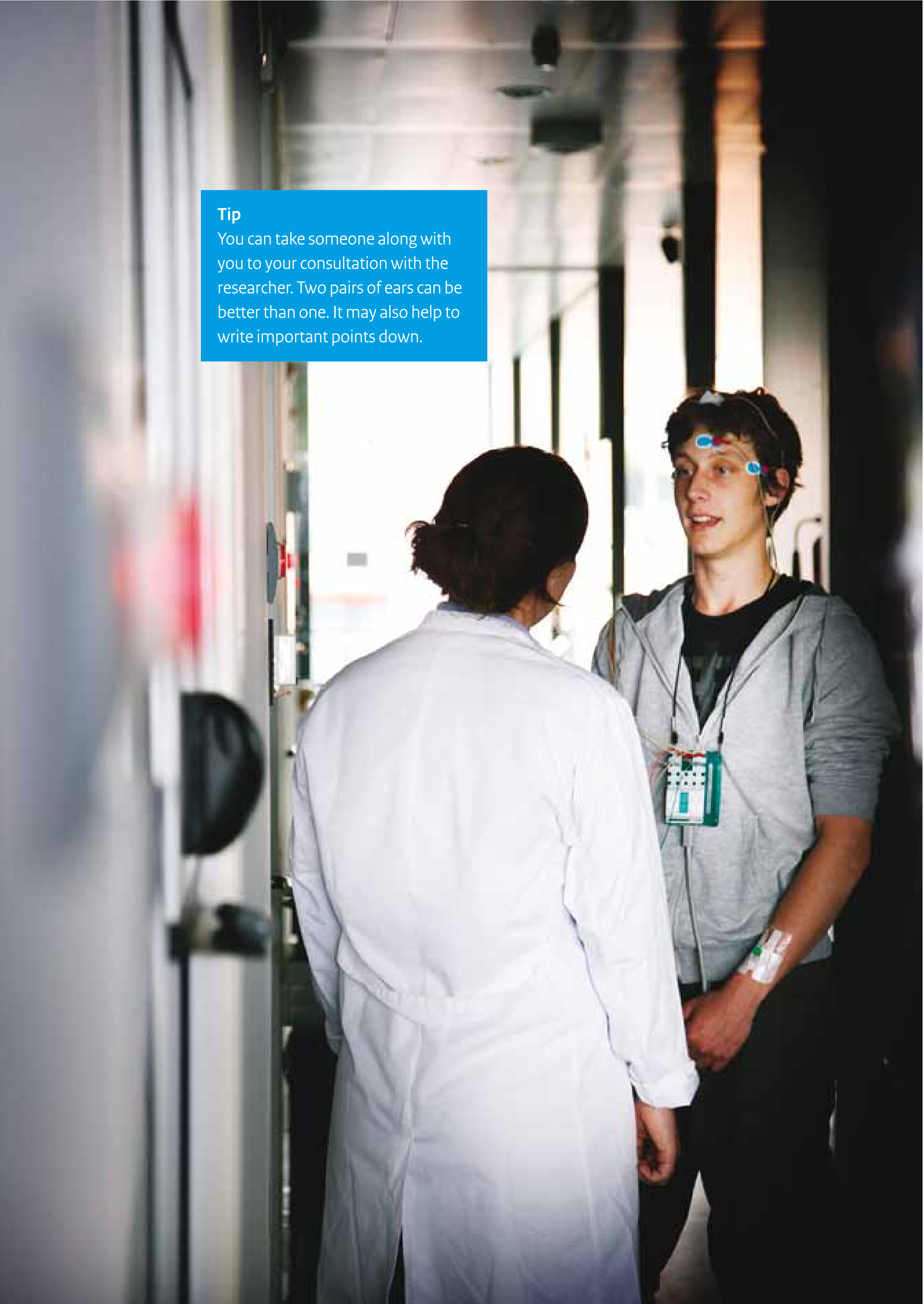

Annex 2: The development of new medications

Around half of all medical research is research on a medication. Before patients can receive a new medication, researchers have to ensure it is safe. A new medication is developed in three steps.

1. **Laboratory**
   In the laboratory researchers constantly work on the development of new medications. This is also where the researchers conduct extensive tests on these medications.
2. **Laboratory animals**
   If the research results are positive, further research with laboratory animals is carried out. The researchers examine the effect of the medication on the animals. They also test whether there are side effects.
3. **Research involving human beings**
   Research with laboratory animals will determine whether the medication seems safe and effective. If so, research with human participants commences. Extensive testing has therefore been carried out before you are asked to participate in a research. Research involving human participants consists of four phases.

   Phase 1 Is the medication safe?
   In the first phase, the researchers examine whether the research participants can tolerate the medication. The research participants in this phase are usually healthy volunteers, though they can also be patients. The researchers also examine how the medication works in the body.
   Phase 2 Does the medication work?
   If the medication is deemed safe it is tested on patients. It is in this phase that the researchers can examine whether it is actually effective in treating the ailment or illness..
   Phase 3 Does the medication work better than existing drugs?
   If the results are positive the researchers introduce more patients to the research. They often compare the medication with an existing drug. If the medication appears to be sufficiently effective it is officially registered as a medication. Doctors may then prescribe the medication.
   Phase 4 What are the long-term effects?
   Studies are also conducted on medications that are already registered. This may involve up to 10,000 patients. The purpose is to examine their long-term effects.

Annex 3: Space for own questions and notes

Colophon
This brochure has been produced for the Ministry of Health, Welfare and Sport in association with the Central Committee on Research involving Human Subjects and the recognised medical ethics review committees.

For a free brochure
Visit the website www.postbus51.nl, or phone 0800 – 8051 (no charge).

Impression: 5,000
Autumn 2009

Appendix 2: trial scheme

Hereby we provide you a schematic overview of what the 5 weeks of the trial will look like for you.

Two to seven days before start of the treatment and at the first day of treatment we will ask you to score the intensity of your itch complaints on a form that will be provided by your physician or research nurse.

The treatment duration is 21 days (3 weeks). On the first and last day of the treatment we ask you to withdraw a vial of blood and complete two questionnaires. In addition, we ask you to keep a diary at home during the 21 days of treatment.

Two weeks after stop of the treatment, on day 35 of the trial, you will come back to the hospital to again score your itch intensity, complete the questionnaires and withdraw another vial of blood. At that time you may also discuss with your physician if further treatment for your itch complaints is needed.

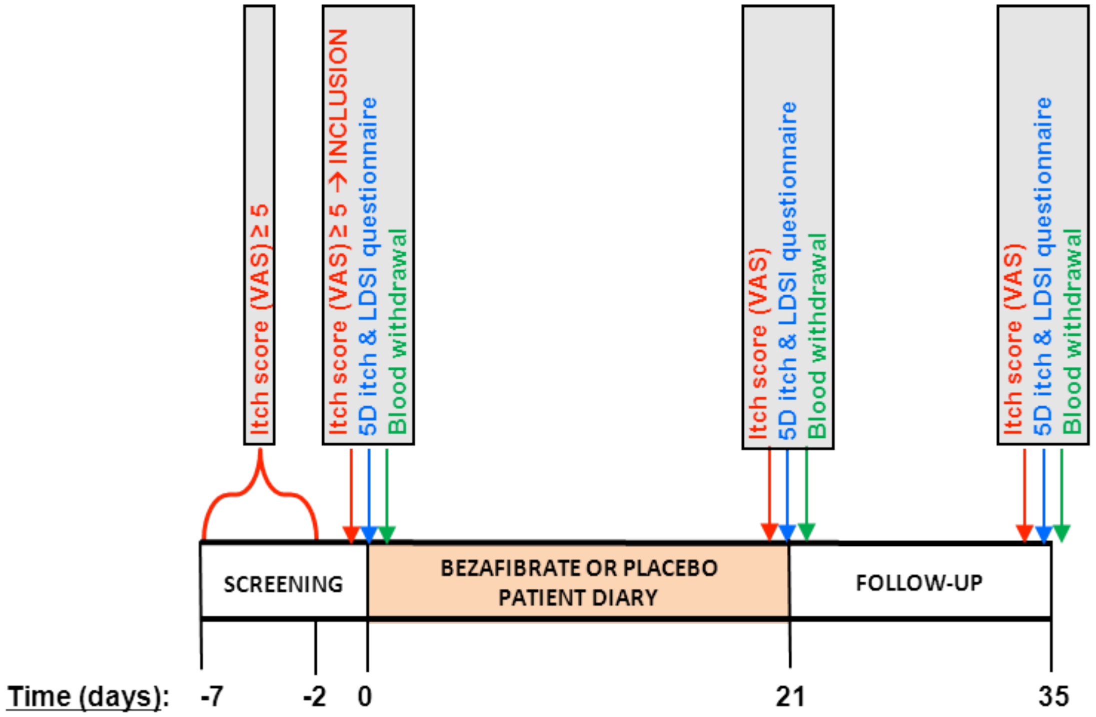

Appendix 3: side effects of study medication

Just as any medication, the medication used in this study may cause side effects. This appendix provides a brief overview of side effects you may expect after taking the study medication. Whenever you suspect one or more side effects, please inform your physician or research nurse.

The following side effects are frequently reported (1-10%, meaning 1 of 10-100 people):

- Loss of appetite

The following side effects are sometimes reported (0,1-1%, meaning 1 of 100-1000 people):

- Hypersensitivity reactions (including anaphylaxis)
- Dizziness*, headache
- Nausea, stomach ache, dyspepsia, constipation, diarrhoea, cholestasis
- Itch, urticarial, skin rash, hypersensitivity to sun light, hair loss
- Muscle weakness, myalgia, muscular spasm
- Acute kidney failure
- Erectile dysfunction
- Disturbed blood tests: increase in CK, creatinine, ALP, transaminases, thrombocytes; decrease in Hb, Ht, white blood cell count, gamma-GT

*Please be aware that dizziness may affect the ability to drive and operate on machinery.

The following side effects are rarely reported (0,1-0,01%, meaning 1 of 1000-10000 people):

- Depression
- Peripheral neuropathy (nerve pain or numbness of fingers and/or toes), paraesthesia (altered pain sensation)
- Pancreatitis

The following side effects are very rarely or incidentally reported (less than 0,01%, meaning 1 of 10000 people):

- Low blood cell count
- Interstitial lung disease
- Bile stones
- Thrombocytopenic purpura, erythema multiforme, Stevens-Johnson syndrome, toxic epidermal necrolysis, rabdomyolysis, increased gamma glutamyl transferase.

Appendix 4: contact information study physicians and nurses

For questions regarding your health in the context of this study you may contact the principal investigator or independent physician in your center:

City	Hospital	Principal investigator	Independent physician
Amsterdam	AMC	Prof. Dr. U. Beuers<br>Tel. 020-5662422<br>u.h.beuers@amc.uva.nl	Drs. S.B. Willemse<br>Tel. 020-5668981<br>s.b.willemse@amc.uva.nl
Amsterdam	VUmc	Dr. D. Ramsoekh<br>Tel. 020-4444304<br>d.ramsoekh@vumc.nl	Dr. C.M.J. van Nieuwkerk<br>Tel. 020-4440613<br>cmj.vannieuwkerk@vumc.nl
Groningen	UMCG	Dr. M. de Vree<br>Tel. 06-55256255<br>j.m.l.de.vree@umcg.nl	Dr. T.C.M.A. Schreuder<br>Tel. 06-55256253<br>t.c.m.a.schreuder@umcg.nl
Maastricht	MUMC	Prof. Dr. P.L.M. Jansen<br>Tel. 043-3876543<br>plm.jansen@maastrichtuniversity.nl	Dr. G.H. Koek<br>Tel. 043-3875021<br>gh.koek@mumc.nl
Rotterdam	Erasmus MC	Dr. H.R. van Buuren<br>Tel. 010-7035942<br>h.vanbuuren@erasmusmc.nl	Dr. R.J. de Knegt<br>Tel. 010-7033041<br>r.deknegt@erasmus.nl
Leiden	LUMC	Prof. Dr. B. van Hoek<br>Tel. 071-5263507<br>b.van_hoek@lumc.nl	Dr. A.E. van der Meulen-de Jong<br>Tel. 071-5263507<br>Ae.meulen@lumc.nl
Nijmegen	Radboud MC	Prof. Dr. J.P.H. Drenth<br>Tel. 024-3613999<br>joostphdrenth@cs.com	Dr. D.J. de Jong<br>Tel. 024-3613999<br>d.dejong@mdl.umcn.nl
Utrecht	UMCU	Dr. K. van Erpecum<br>Tel. 088-7555555<br>k.j.vanerpecum@umcutrecht.nl	Drs. J.F. Monkelbaan<br>Tel. 088-7555555<br>j.f.monkelbaan@umcutrecht.nl

For questions regarding the study itself you may contact the coordinating investigator:

Drs. Ruth Bolier, arts-onderzoeker
Academic Medical Center (AMC), Amsterdam
Meibergdreef 69-71
1105 BK Amsterdam
a.r.bolier@amc.nl
Tel. 020-5668701

Appendix 5: Insurance information for study subjects

The AMC has insurance for all who participate in this study. This insurance covers damage resulting from participation in this study. Damage may be caused during the study or within 4 years after its end. You are required to declare any damage within 4 years to the insurance company.

Not all damage is covered by the insurance. Below you will find information on what damage is not covered. These conditions are defined in the Insurance Decree of the Central Committee on Research Involving Human Subjects (see www.ccmo.nl).

In case of damage you may directly contact the insurance company:

The insurance company of this study is:	
Name:	Onderlinge Waarborgmaatschappij Centramed B.A.
Address:	Appelgaarde 4, 2272 TK Voorburg
Phone:	070-3017070

De insurance covers maximally €650.000,- per study subject and maximally €5.000.000,- for the entire study.

The following damage is **not** covered:

- damage you were informed about to risk by participation to this study. This does not count in case the damage is worse than was anticipated on or if the risk was very low
- health damage that would have happened as well if you wouldn't have participated in the study
- damage that was caused by not (completely) following the instructions
- damage to your progeny, as a consequence of a negative outcome of the study to you or your progeny
- damage due to existing treatment methods in case of studies including existing treatment methods

Informed consent form FITCH trial

- I have read the information letter (version 3.0 dd 20-01-2016). I was able to ask questions. My questions have been answered adequately. I have had enough time to decide about participation in this trial
- I am aware that participation is entirely voluntary. I know that at any time during the study, without giving a reason, I can withdraw from participation.
- I agree to inform my general physician and other medical specialists about my participation
- I am aware that some people have access to my personal data. These people are named in the information letter.
- I agree to use my personal data for the aims mentioned in the information letter
- I agree to save my personal data during 15 years after finishing the trial.
- I do/ I do not* agree to save my blood during 5 years after finishing the trial, so that it may be used for additional research as described in the patient information letter
- I do/ I do not* allow the investigator to approach me for follow-up research
- I agree to participate in this trial

Name **participant**: Date of birth: __ / __ / __

Signature: Date: __ / __ / __

Hereby I declare that I have informed the participant about the trial. If at any time during the trial information will be available that may affect the will to participate I will inform him/her in time. After signing, I will give a copy of this form to the patient.

Name **investigator** (or a representative, e.g. physician/ research nurse):

Signature: Date: __ / __ / __

----

* Please indicate what is applicable
