# Supplementary material for: Fibrates for the treatment of cholestatic itch (FITCH): study protocol for a randomized controlled trial
Source: Trials. 2017 May 23;18:230. doi: 10.1186/s13063-017-1966-8 (PMC5442649; doi:10.1186/s13063-017-1966-8)
Supplement: Supplementary file 3 — Flow diagram of the FITCH study protocol. Abbreviations: 5D five-dimensional, LDSI Liver Disease Symptom Index, VAS Visual Analogue Scale. (PDF 49 kb) [file 13063_2017_1966_MOESM3_ESM.pdf]

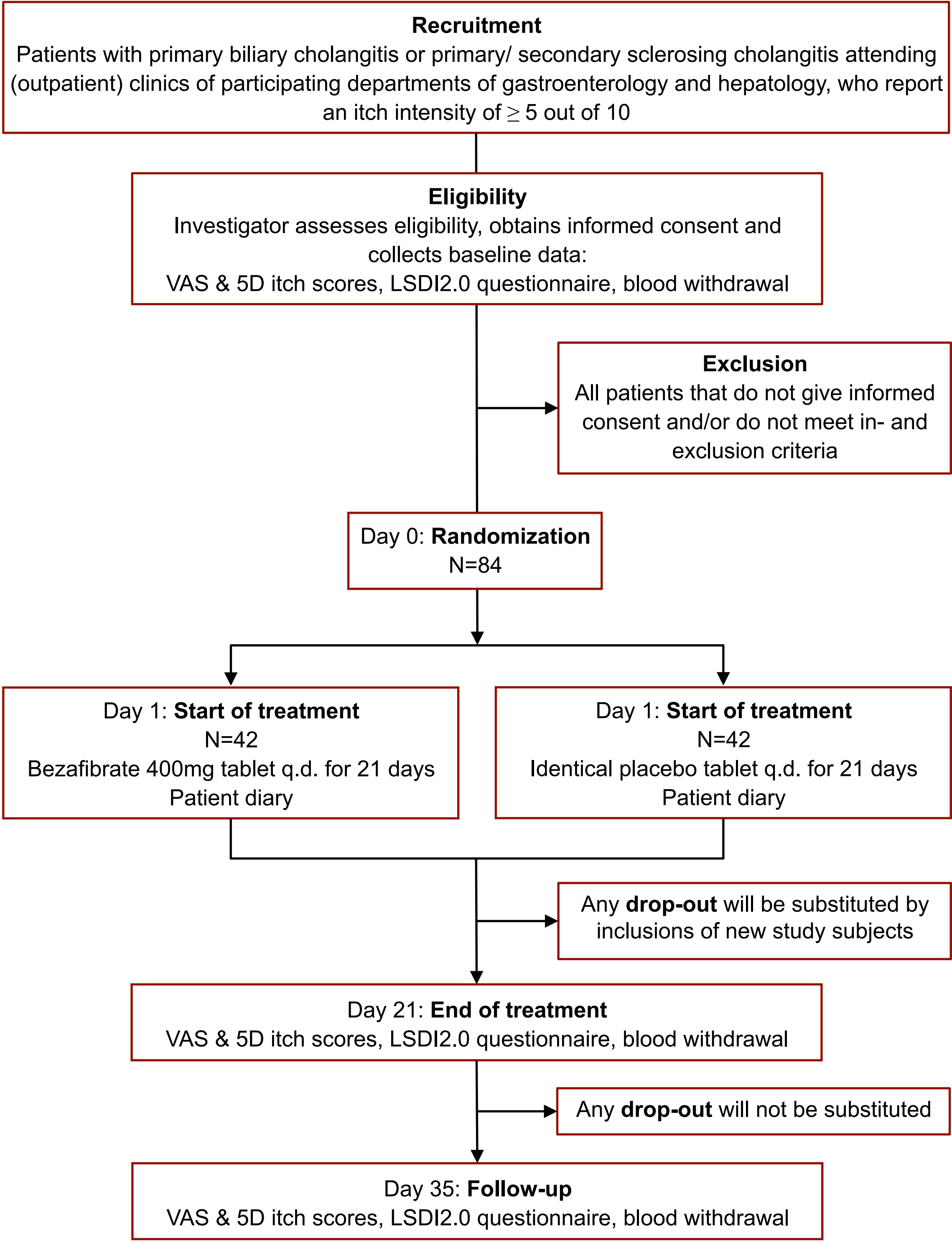
Recruitment
Patients with primary biliary cholangitis or primary/ secondary sclerosing cholangitis attending (outpatient) clinics of participating departments of gastroenterology and hepatology, who report an itch intensity of ≥ 5 out of 10
Eligibility
Investigator assesses eligibility, obtains informed consent and collects baseline data:
VAS & 5D itch scores, LSDI2.0 questionnaire, blood withdrawal
Exclusion
All patients that do not give informed consent and/or do not meet in- and exclusion criteria
Day 0: Randomization
N=84
Day 1: Start of treatment
N=42
Bezafibrate 400mg tablet q.d. for 21 days
Patient diary
Day 1: Start of treatment
N=42
Identical placebo tablet q.d. for 21 days
Patient diary
Any drop-out will be substituted by inclusions of new study subjects
Day 21: End of treatment
VAS & 5D itch scores, LSDI2.0 questionnaire, blood withdrawal
Any drop-out will not be substituted
Day 35: Follow-up
VAS & 5D itch scores, LSDI2.0 questionnaire, blood withdrawal
